# Temporomandibular joint damage in K/BxN arthritic mice

**DOI:** 10.1038/s41368-019-0072-z

**Published:** 2020-02-06

**Authors:** Sabine Kuchler-Bopp, Alexandre Mariotte, Marion Strub, Chrystelle Po, Aurore De Cauwer, Georg Schulz, Xavier Van Bellinghen, Florence Fioretti, François Clauss, Philippe Georgel, Nadia Benkirane-Jessel, Fabien Bornert

**Affiliations:** 1 INSERM (French National Institute of Health and Medical Research), UMR 1260, Regenerative NanoMedicine (RNM), FMTS, Strasbourg, France; 2 INSERM, UMR 1109, Immuno Rhumatologie Moléculaire (IRM), FMTS, Strasbourg, France; 3 Fédération Hospitalo-Universitaire (FHU) OMICARE, Université de Strasbourg, Strasbourg, France; 4 Faculté de Chirurgie Dentaire, Université de Strasbourg (UDS), 8 rue Ste Elisabeth, Strasbourg, France; 5 Pôle de Médecine et Chirurgie Bucco-Dentaires, Hôpitaux Universitaires de Strasbourg (HUS), 1 place de l’Hôpital, Strasbourg, France; 6 ICube UMR 7357, Université de Strasbourg, CNRS, FMTS, 4 rue Kirschleger, Strasbourg, France; 7 Department of Biomedical Engineering, Core Facility Micro- and Nanotomography, Biomaterials Science Center (BMC), University of Basel, Gewerbestrasse 14 4123, Allschwil, Switzerland

**Keywords:** Rheumatoid arthritis, Animal disease models

## Abstract

Rheumatoid arthritis (RA) is an autoimmune disease affecting 1% of the world population and is characterized by chronic inflammation of the joints sometimes accompanied by extra-articular manifestations. K/BxN mice, originally described in 1996 as a model of polyarthritis, exhibit knee joint alterations. The aim of this study was to describe temporomandibular joint (TMJ) inflammation and damage in these mice. We used relevant imaging modalities, such as micro-magnetic resonance imaging (μMRI) and micro-computed tomography (μCT), as well as histology and immunofluorescence techniques to detect TMJ alterations in this mouse model. Histology and immunofluorescence for Col-I, Col-II, and aggrecan showed cartilage damage in the TMJ of K/BxN animals, which was also evidenced by μCT but was less pronounced than that seen in the knee joints. μMRI observations suggested an increased volume of the upper articular cavity, an indicator of an inflammatory process. Fibroblast-like synoviocytes (FLSs) isolated from the TMJ of K/BxN mice secreted inflammatory cytokines (IL-6 and IL-1β) and expressed degradative mediators such as matrix metalloproteinases (MMPs). K/BxN mice represent an attractive model for describing and investigating spontaneous damage to the TMJ, a painful disorder in humans with an etiology that is still poorly understood.

## Introduction

The upper part of the temporomandibular joint (TMJ) is formed by the temporomandibular fossa or glenoid fossa, and the lower part is formed by the mandibular condyle. An ellipsoidal articular disc composed of fibrous connective tissue divides the joint into two parts: the upper and the lower articular cavities. These cavities are limited by the synovial membrane and filled with fluid. Chronic joint diseases, most often those affecting the limb joints, can also affect the temporomandibular joint, causing cartilage degradation, subchondral bone degradation (erosion and resorption), sclerosis, and a reduction in the joint space. In the case of severe condyle destruction, malocclusion (defined as an abnormal disposition of the teeth in the jaw, generating teeth and jaw pain), and sometimes vertebral damage may occur.^1,2^

Rheumatoid arthritis (RA) is an autoimmune disease characterized by chronic articular inflammation (driving swelling, stiffness, and pain) and sometimes accompanied by extra-articular manifestations. Affecting nearly 1% of the population worldwide, RA is associated with comorbidities (such as cardiovascular disorders). Inflammation in RA is orchestrated by a complex interplay between several types of immune cells (T and B cells and macrophages) and stromal cells, such as fibroblast-like synoviocytes (FLSs). FLSs (or type B synoviocytes) are mesenchymal resident cells that have been described in recent decades as active players in cartilage damage through proliferative/invasive properties (so-called “pseudo-tumoural” behavior) and the secretion of inflammatory (e.g., interleukin-6 (IL-6)) and degradative (e.g., matrix metalloproteases (MMPs)) mediators.^3^

While RA typically affects the small peripheral joints (metacarpo-/metatarsophalangeal and proximal interphalangeal joints and wrists), TMJ involvement has also been reported, and some patients may experience chronic pain, limitation of mouth opening, and mandible deviation, especially in juvenile types.^4,5^ Different models of osteoarthritis of the TMJ have been described.^6–9^ However, despite the significant number of murine (poly)-arthritis models reported in the literature, few reliable TMJ arthritis animal models are currently available and have been extensively described.

The K/BxN mouse model of polyarthritis used in this work was originally described in 1996.^10^ K/BxN mice were obtained by crossing KRN transgenic animals with non-obese diabetic mice (NOD). They develop severe inflammatory arthritis of the leg joints and bone destruction without sexual dimorphism.^10^ This widely described model is notably used to trigger rapid and consistent joint inflammation in healthy mice by transferring serum harvested from K/BxN animals, which contains high levels of autoantibodies.^11^ IL-6 and TNF-α, which are targeted by reference biodrugs in RA patients, are also overexpressed in the joints of K/BxN mice.^12^ Finally, the K/BxN model was chosen for this work because of its clinical relevance for human RA; it is spontaneous and progressive, evolves to chronicity, and exhibits symmetrical distribution and joint destruction.^12^

In RA patients, the degree of arthritis is assessed through conventional radiography, cone beam multi-slice computed tomography (CBCT or MSCT), and magnetic resonance imaging (MRI).^1,13,14^ The dose of X-ray delivered during CBCT is low, which is advantageous for exploring TMJ in children, and enables high spatial resolution with limited irradiation levels.^15–18^ The advantage of CBCT is the visualization of dense structures, such as the temporal bone and mandibular condyle, in three dimensions. Magnetic resonance imaging seems to be the most relevant imaging modality for assessing TMJ osteoarthritis, as early changes can be detected, and both hard and soft tissues and even joint fluid can be visualized.^13,19^ Gradient-echo T1 sequences enable evaluation of bone changes, while gradient-echo T2 sequences are mainly used to analyze less dense structures such as cartilage, joint disc, or synovial fluid in the upper and lower joint cavities.^20^

The aim of this study was to describe TMJ inflammation and damage in an arthritis mouse model in comparison with that in healthy controls by using histological and imaging techniques already used in clinical practice.

## Results

### Characterization of K/BxN mice

Several murine arthritis models have been reported in the literature.^21^ While defects of the limb joints have been extensively described, potential TMJ disease was addressed in only a few of these models (listed in Supplementary Table 1). In this work, we decided to use the K/BxN model, which exhibits arthritis that is highly penetrant and reproducible and typically displays progressive, chronic, severe, and deforming symmetrical arthritis. Offspring of KRN-transgene-bearing mice develops clinical arthritis manifestations from 5 weeks of age without any sexual dimorphism. In this study, we chose animals that were 6–8 months old and displayed markedly progressive ankylosis accompanied by joint swelling and limb deformities (Supplementary Fig. 1a). We quantified serum IL-6 in K/BxN mice by enzyme-linked immunosorbent assay (ELISA). The parental mouse lines NOD and ICR (imprinting control region) were used as controls (Supplementary Fig. 1b). IL-6 secretion was significantly enhanced in the blood of K/BxN mice compared with the blood of control mice (Supplementary Fig. 1b), in agreement with systemic inflammation in these animals. IL-1β and TNF-α levels were, however, below the detection threshold.

As this model is known to experience destructive limb joint damage, we assessed arthritis-associated histomorphological changes in the knee joint. Micro-computed tomography (μCT) examination showed clear differences in bone density (indicative of erosion) in the knees of K/BxN mice, while control animals appeared unaffected (Supplementary Fig. 1c–g); subchondral bone changes were also visible in 3D reconstructions (Supplementary Fig. 1d–h). In K/BxN mice, hematoxylin–eosin staining further revealed cartilage matrix discontinuity with remnant cartilage and residual bone-adopting irregular structures; this was accompanied by knee joint inflammation (Supplementary Fig. 1i). These observations are in sharp contrast with those found in control mice, in which the articular cavity was acellular and the cartilage appeared smooth and uniform (Supplementary Fig. 1e).

Furthermore, staining of cartilage-specific sulfated mucopolysaccharides with alcian blue (used at pH = 1) showed that K/BxN cartilage was markedly eroded (Supplementary Fig. 1j) compared with control cartilage (Supplementary Fig. 1f). Together, our observations confirm that the limb joints of K/BxN mice exhibit arthritis with destructive and cartilage-erosive features.

### Histological and morphological analyses revealed previously unsuspected TMJ cartilage damage

**Fig. 1:**
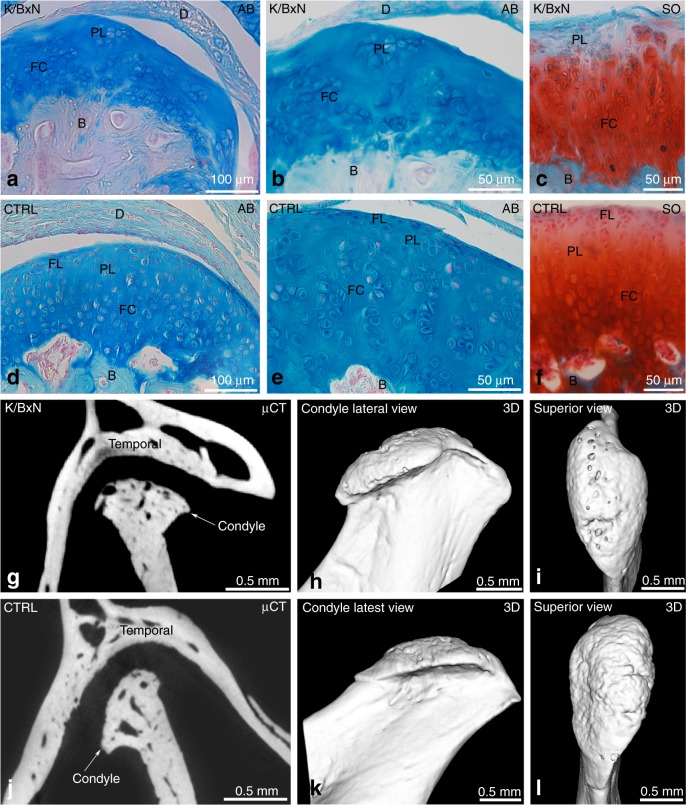
Histological and morphological analyses of K/BxN and control TMJs. Histology of the temporomandibular joint (TMJ) of an 8-month-old K/BxN mouse (**a**–**c**) and a control mouse (**d**–**f**) after alcian blue staining (**a**, **b**, **d**, **e**) and Safranin O staining (**c**, **f**). **g**, **j** Micro-computed tomography (μCT) sections of the TMJ of an 8-month-old K/BxN mouse (**g**) and a control mouse (**j**). (**h**, **i**, **k**, **l**) 3D reconstructions of the condyles of an 8-month-old K/BxN mouse (**h**, **i**) and a control mouse (**k**, **l**). B, bone; D, disc; FC, fibrocartilage; FL, fibrous layer; PL, proliferative layer

We next considered potential TMJ alterations. First, we performed histological analyses (alcian blue, pH = 1, to specifically stain mucopolysaccharides coupled with fast red to stain nuclei) on TMJ sections to assess cartilage integrity in K/BxN mice and controls (Fig. 1a, b, d, e and Supplementary Fig. 2). We also performed safranin O staining to reveal proteoglycans in cartilage tissue coupled with staining with fast green, an acidic dye that reacts with cytoplasmic proteins (Fig. 1c, f). As shown in Fig. 1a, d, the articular disc (D) separated the articular cavity into the upper and lower parts. We first observed a discontinuity in the fibrous layer (FL), suggesting degradation of the extracellular matrix; the cartilage thickness also seemed to be reduced in the TMJ of K/BxN mice (Fig. 1a–c and Supplementary Fig. 3b–f), whereas the subchondral bone remained unaffected. The thickness of the cartilage was evaluated and determined to be (56 ± 4) μm in control mice and (54 ± 12) μm in K/BxN mice. Control mouse cartilage appeared intact (Fig. 1d–f). We then used μCT and compared the temporal surface and the mandibular condyle of the TMJ of 6- to 8-month-old K/BxN mice (Fig. 1g and Supplementary Fig. 3b–f) with those of TMJs harvested from age-matched control mice (Fig. 1j and Supplementary Fig. 3a). Three-dimensional isosurface reconstructions were made from the DICOM dataset. Different bone abnormalities (erosion) were detected in the TMJ of K/BxN mice (Fig. 1h, i and Supplementary Fig. 3b–f) in comparison with the TMJ of controls (Fig. 1k, l and Supplementary Fig. 3a). Subchondral bone damage (subchondral cyst) was also visible in microtomography slices. These observations indicate that K/BxN mice also exhibit TMJ defects that include cartilage thinning and reduced matrix proteoglycans.

### The superficial cartilage layer of the TMJ is specifically altered in K/BxN mice

**Fig. 2:**
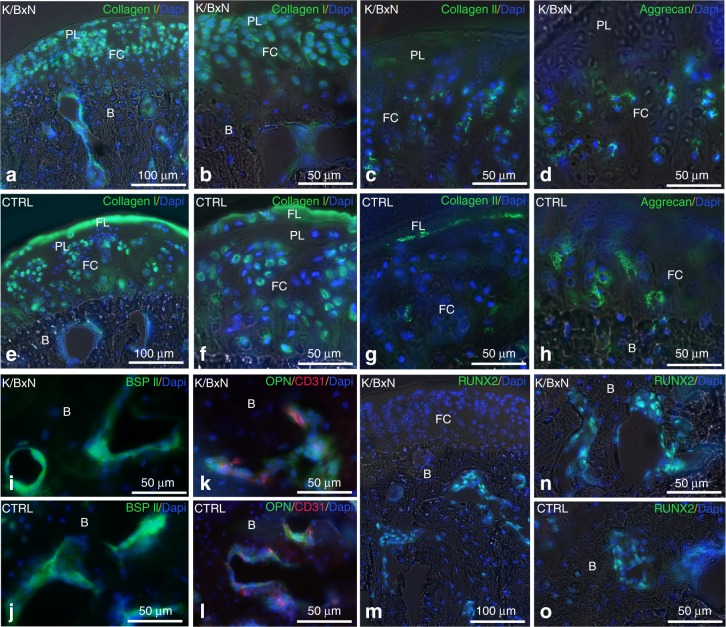
Immunolocalization of specific cartilage and bone proteins in K/BxN and control TMJs. Immunofluorescence for Collagen I (**a**, **b**, **e**, **f**), Collagen II (**c**, **g**), Aggrecan (**d**, **h**), BSPII (**i**, **j**), Osteopontin and CD31 (**k**, **l**), and RUNX2 (**m**–**o**) in cryostat sections of a K/BxN TMJ (**a**–**d**, **i**, **k**, **m**, **n**) and a control TMJ (**e**–**h**, **j**, **l**, **o**)

Having shown that K/BxN mice display anatomical alterations in TMJ cartilage, we next monitored the expression of markers by immunofluorescence in these animals. In the control TMJ, type I collagen (Col-I) was expressed in the fibroblasts of the fibrous layer (FL), in undifferentiated mesenchymal cells of the proliferative layer (PL), in chondrocytes of the fibrocartilage (FC) of the mandibular control condyle, and in osteoblasts (Fig. 2e, f). In K/BxN, Col-I was expressed in the proliferative layer, the fibrocartilage, and bone, while its expression was no longer detected in the fibrous layer (Fig. 2a, b). Type II collagen (Col-II) expression in the fibroblasts of the fibrous layer and in chondrocytes of the fibrocartilage was globally weaker than Col-I expression in the control mandible (Fig. 2g), but similarly disappeared in the fibrous layer cartilage of K/BxN mice (Fig. 2c).

Aggrecan, the expression of which was restricted to fibrocartilage, was unchanged between diseased and healthy animals (Fig. 2d, h). We also tested the expression of bone-specific proteins, such as bone sialoprotein II (BSPII) (Fig. 2i, j), osteopontin (OPN) (Fig. 2k, l), the bone-specific transcription factor RUNX2 (Fig. 2m–o), and the vascular endothelial marker CD31 (Fig. 2k, l). BSPII and OPN were detected in the cytoplasm of osteocytes (Fig. 2i–l), while RUNX2 exhibited nuclear localization (Fig. 2m–o); CD31^+^ endothelial cells were found close to osteocytes (Fig. 2k, l). However, we did not observe any difference between control and K/BxN TMJs by using these markers. These data suggest that TMJ defects observed in K/BxN mice (Fig. 1) are restricted to the superficial cartilage and are characterized by a significant reduction in fibrous layer matrix proteins.

### Synovial fluid expansion can be observed in K/BxN TMJs

**Fig. 3:**
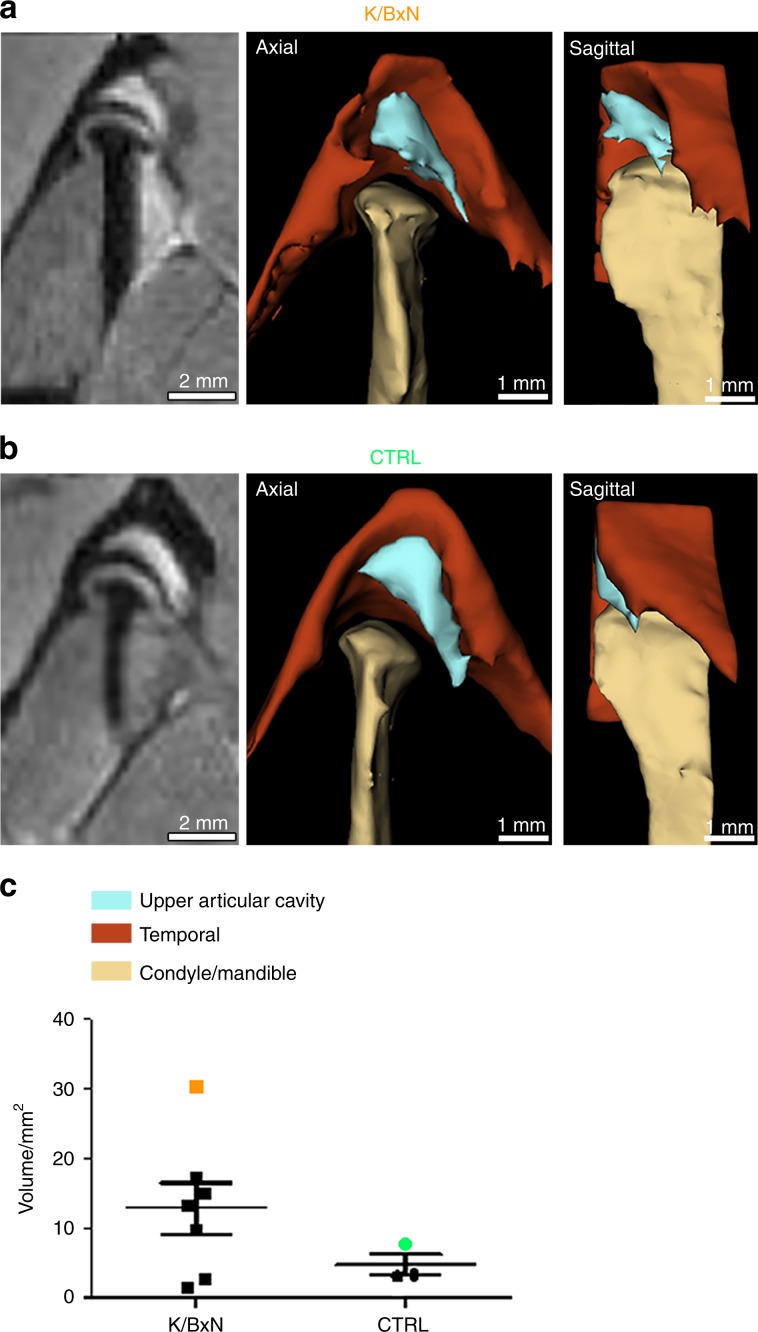
Comparison of synovial fluid volume of K/BxN and control TMJs. Magnetic resonance imaging (MRI) and 3D reconstructions of the TMJ of a K/BxN mouse (**a**) and a control mouse (**b**). **c** Volume measurement of the upper articular cavity of seven K/BxN mice and three control mice. The orange square represents the K/BxN mouse shown in **a**, and the green circle represents the control mouse shown in **b**

We also used magnetic resonance imaging (MRI) with T2-weighted imaging to visualize liquid in the TMJ of control and arthritic mice. In these settings (Fig. 3a, b and Supplementary Fig. 4), bone structure such as that of the mandibular condyle (shown in yellow in the reconstruction pictures) and temporal bone (red) were observed to have a low signal intensity; conversely, liquids, such as synovial fluid (blue) that was present in the upper and lower cavities, were associated with hypersignals in T2 based on water content. The articular disc was observed to have intermediate signal intensity. After 3D reconstruction, the mean volume of the synovial fluid present in the upper articular cavity (blue) was increased in several K/BxN mice (Fig. 3a and Supplementary Fig. 4) compared with control mice (Fig. 3b). The total volume of the upper articular cavity was enhanced in the K/BxN TMJ (+253.5%), albeit without reaching statistical significance (Fig. 3c). We observed heterogeneous volumes of the upper articular cavity in K/BxN mice, whereas this measure was homogeneous in control mice (Fig. 3c and Supplementary Fig. 4). Four K/BxN mice exhibited increased TMJ articular volume; in the three others, this volume was comparable to that of the controls (Fig. 3c and Supplementary Fig. 4), suggesting that synovial fluid expansion, a feature found in various human arthropathies, may occur in some mice but not others.

### Characterization of the fibroblast-like synoviocytes (FLSs) from K/BxN and control TMJs

**Fig. 4:**
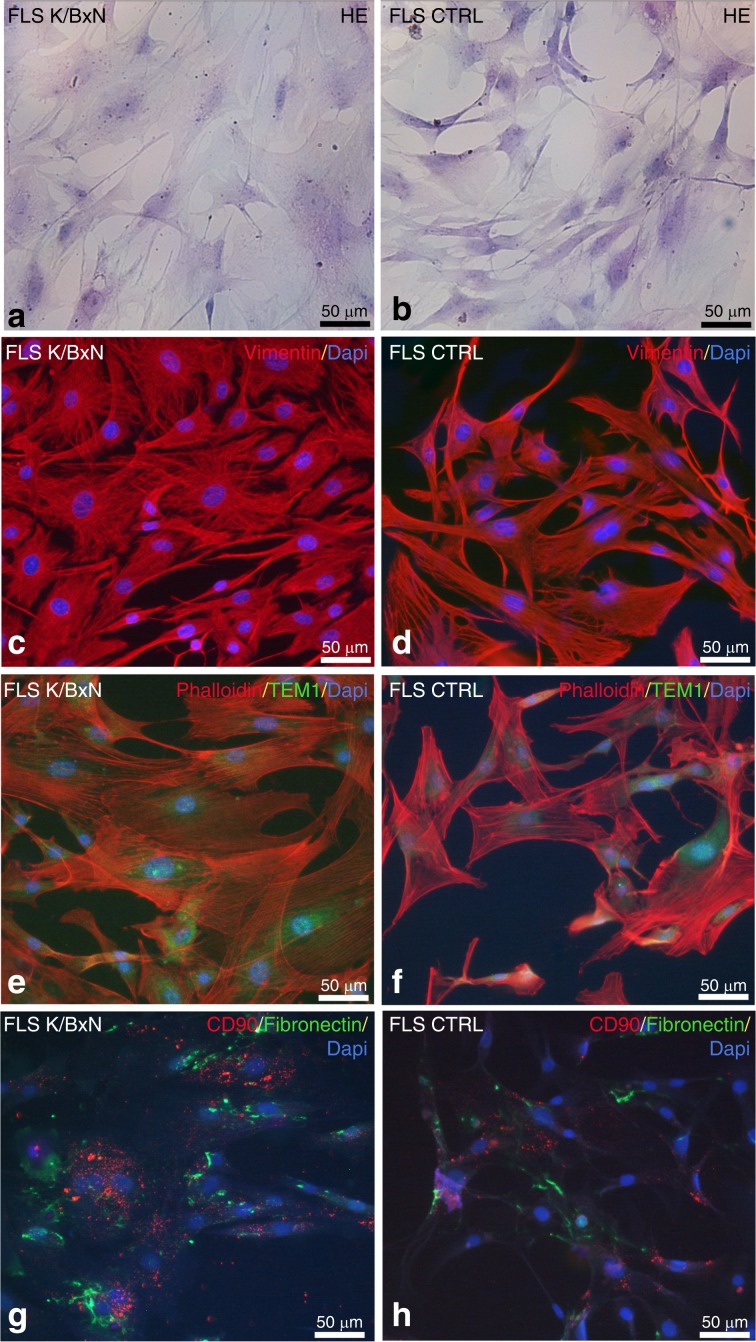
Characterization of fibroblast-like synoviocytes (FLS) from K/BxN and control TMJs. Characterization of fibroblast-like synoviocytes (FLSs) from K/BxN TMJs (**a**, **c**, **e**, **g**) and control TMJs (**b**, **d**, **f**, **h**) by using hematoxylin–eosin staining (**a**, **b**) and immunofluorescence for vimentin (c, d), phalloidin and TEM1 (**e**, **f**), and CD90 and fibronectin (**g**, **h**). Nuclei were stained with 4′,6-diamidino-2-phenylindole (DAPI)

Fibroblast-like synoviocytes (FLSs) are resident joint stromal cells that express mesenchymal markers. They are essential for maintaining homeostatic functions under noninflammatory conditions, such as the synthesis of the synovial fluid components proteoglycans and lubricin.^22^ In contrast, FLSs are active contributors to synovial inflammation and cartilage damage in the context of rheumatoid arthritis.^23^ Our observations of TMJ cartilage alterations in K/BxN mice prompted us to analyze the functional properties of TMJ FLSs in more detail. For this, we first derived and cultured primary FLSs from TMJs dissected from K/BxN and control mice and characterized them at passage 3. When confluent, FLSs exhibited a classic spindle-shaped fibroblastic phenotype after hematoxylin–eosin staining (Fig. 4a and b). Non-inflamed and K/BxN FLSs stained positively for the stromal mesenchymal markers vimentin and fibronectin (Fig. 4c, h) and the synovial fibroblastic surface markers CD90 (Thy-1) (Fig. 4g, h) and TEM1 (Fig. 4e, f). Interestingly, immunofluorescence signals for TEM1, CD90, and fibronectin appeared to be increased in K/BxN FLSs compared with control FLSs.

**Fig. 5:**
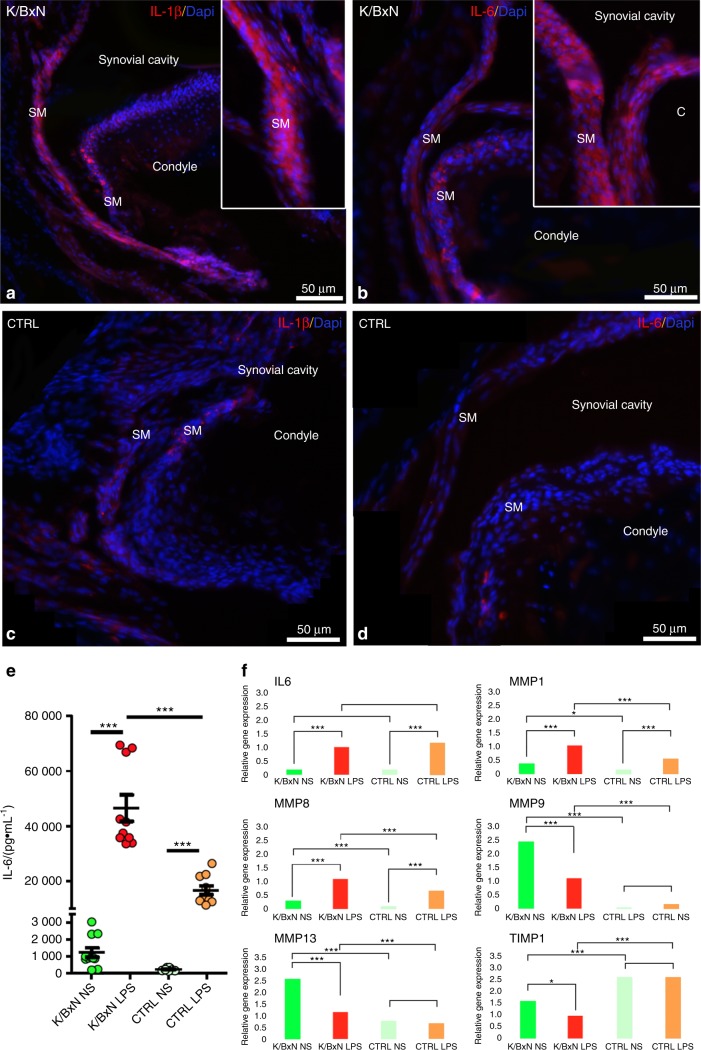
Expression of inflammatory cytokines and matrix metalloproteinases in K/BxN and control FLS. Immunofluorescence for IL-1β (**a**, **c**) and IL-6 (**b**, **d**) in sagittal sections of the TMJ of K/BxN mice (**a**, **b**) and control mice (**c**, **d**). (**e**) Quantification of IL-6 (pg·mL^−^^1^) in the culture medium of unstimulated (NS) K/BxN and control TMJ FLSs and those stimulated with LPS for 24 h. (**f**) RT-qPCR experiments to monitor *Il-6*, *Mmp1*, *8*, *9,* and *13* and *Timp1* expression in K/BxN and control FLSs treated with or without LPS for 24 h. The values are the means ± SEMs; *P < 0.01, ***P < 0.000 1

FLSs were then stimulated for 24 h with 1 μg·mL^−^^1^ LPS from *Escherichia coli*, and we monitored IL-6 production by ELISA (Fig. 5e). We observed increased basal (unstimulated conditions) IL-6 production in K/BxN FLSs, and this difference was reinforced after LPS treatment (Fig. 5e). In contrast, no IL-1β production was detected in K/BxN FLSs stimulated with or without LPS (not shown). We also assessed *Il-6*, *Mmps* (*Mmp1, 8, 9, 13*), and *Timp1* gene expression by RT-qPCR in FLSs treated with or without LPS for 24 h (Fig. 5f). *Il-6* transcripts were significantly enhanced in FLSs after LPS treatment, but no difference was observed between K/BxN and control FLSs. Conversely, we observed enhanced *Mmp1* and *Mmp8* expression in K/BxN and control FLSs after LPS treatment, with significantly increased expression in K/BxN FLSs compared with control FLSs. In contrast, the expression of *Mmp9* and *Mmp13* was significantly reduced after LPS treatment, but remained significantly more elevated in K/BxN FLSs than in control FLSs. Finally, the expression of *Timp1* was reduced in K/BxN FLSs treated with or without LPS compared with control FLSs, suggesting decreased or defective MMP inhibition in K/BxN FLSs. In parallel, immunostaining in TMJ sections showed increased IL-6 and IL-1β expression in the synovial membrane (SM) of K/BxN mice but not control mice, revealing increased local inflammation (Fig. 5a–d). Inflammation of the synovial membrane is often accompanied by the infiltration of mononuclear cells (lymphocytes and monocytes/macrophages). The cells positive for IL-1β in the synovial membrane of K/BxN (Fig. 5a) were most likely mononuclear cells. Altogether, our data demonstrated that, similar to limb joints, FLSs isolated from the TMJ of K/BxN animals are characterized by an aggressive (pro-inflammatory) phenotype, which likely contributes to the damage also seen in this specific anatomical location.

## Discussion

Rheumatoid arthritis typically affects the small joints, especially those of the hands and feet, but damage to other locations has also been reported. Vertebral and TMJ damage with bone erosion, pain, and decreased jaw function have been described, and the latter may affect nearly 30%–50% of RA patients.^12,24,25^ However, such alterations have been rarely reported in polyarthritis mouse models so far. To our knowledge, morphological observations (using histology and micro-computed tomography (μCT)) of TMJ damage were also made in a TNF-α transgenic model (see Supplementary Table 1).^26^

In this work, we describe for the first time TMJ damage in the K/BxN model. We observed erosive changes that occurred mainly in the superficial parts of the articular cartilage, resulting in the disappearance of the TMJ cartilage fibrous layer. In the peculiar case of the TMJ, the articular surface is not covered with hyaline cartilage, but is rather covered with a fibrous layer characterized by abundant type I collagen and reduced collagen II.^27^ Here, we noticed a dramatic loss of both collagen types together with a paucity of alcian blue staining in K/BxN TMJ sections, indicating the absence of the fibrous layer and associated matrix proteins in the condylar surface. TMJ cartilage damage was, however, less pronounced than that seen in the knee joints, as evidenced by μCT. Many authors have reported that X-ray imaging and especially CBCT is a very effective technique for characterizing TMJ osteoarthritis in humans,^15–17^ and several criteria have been defined: (i) erosion with an interruption or absence of cortical lining, (ii) sclerosis with increased density of cortical lining or subchondral bone, (iii) osteophytes with marginal bone outgrowth, (iv) geodes or subchondral cysts with single or multiple pyriform-shaped subchondral lesions with sclerotic margins, and (v) joint space narrowing.^28^

In this study, we also examined the deeper cartilage layers, such as the fibrocartilage layer, which is schematically subdivided into proliferative and hypertrophic areas.^29,30^ The proliferative area is known as a cell reservoir rich in type I collagen, and the hypertrophic area is enriched in aggrecan-positive chondrocytes.^31^ Aggrecan, the major proteoglycan of articular cartilage, is a multimodular molecule expressed by chondrocytes. It plays an important role in mediating chondrocyte–chondrocyte and chondrocyte–matrix interactions through its ability to bind hyaluronan.^32^ We did not observe any variation in aggrecan expression in fibrocartilage chondrocytes between K/BxN and control mice.

Furthermore, we did not observe any difference in the expression of several bone markers (BSPII, OPN, and RUNX2) or CD31, an endothelial cell marker, indicating that damage only occurred at the superficial part of TMJ cartilage, with the bone and deep cartilage layers remaining intact.

To explain these results, we performed magnetic resonance imaging with T2-weighted imaging of TMJs to detect the presence of ongoing inflammation or synovial fluid expansion. Interestingly, we noted that compared with control mice, K/BxN mice exhibited an important variability in synovial volume. The difference, however, did not reach statistical significance. This observation, which suggests increased volume of the upper articular cavity, is indicative of an inflammatory process, a phenomenon that is either inconsistent between individuals or temporally discontinuous in K/BxN mice. Another explanation for this slight difference could be that the TMJ is relatively enclosed by bone walls formed by the mandibular fossa of the temporal bone, which restricts synovial fluid expansion. Interestingly, these data are in line with observations of human patients, who sometimes display such variability in synovial fluid volume.^33–35^

Because FLSs are active players that contribute to cartilage damage in RA, we considered their potential role in the erosive process observed in the TMJ of K/BxN mice. We obtained and cultured primary FLSs from TMJs by using a protocol adapted from one that is usually used in our laboratory to harvest FLSs from limb joints.^36^ We characterized these cells for their expression of stromal markers, as previously described.^37^ Indeed, the expression of CD90, CD248, fibronectin, and vimentin, together with a fibroblastic phenotype, clearly qualifies our TMJ FLSs as bona fide FLSs, as defined by Hardy et al.^38^ Interestingly, K/BxN TMJ-derived FLSs spontaneously produced more IL-6 than TMJ FLSs derived from control mice, although this difference was not observed at the RNA level, suggesting post-transcriptional regulation. This is in line with our in vivo data, which showed increased serum IL-6 in K/BxN mice, indicating sustained activation of synovial FLSs and macrophages, which are responsible for systemic inflammation. Following lipopolysaccharide (LPS) stimulation, TMJ FLSs from K/BxN mice produced higher amounts of IL-6 than that produced by control cells. In addition, our RT-qPCR experiments revealed LPS-dependent upregulation of various matrix metalloproteinase (MMP) genes in K/BxN FLSs compared with control FLSs; conversely, the expression of *Timp1*, which encodes an inhibitor of MMPs, was downregulated. Altogether, our data link the pro-inflammatory and pro-degradative functions of K/BxN TMJ-derived FLSs to the cartilage alterations that we observed in these arthritic mice. Interestingly, TMJ damage was not observed upon K/BxN serum transfer, an acute arthritis model^11^ (see Supplementary Table 1), suggesting that alterations in this articulation require longer exposure to autoantibodies, such as anti-glucose-6-phosphate isomerase autoantibodies, as described by Tanaka-Watanabe et al.^39^

## Conclusion

Ultimately, this work expands the applicability of K/BxN mice beyond a limb-restricted polyarthritis model. Using several relevant imaging techniques, we described spontaneous TMJ damage in which a local inflammatory process can engage superficial cartilage stripping, ultimately leading to condyle surface erosion. To our knowledge, this is the most extensive description of TMJ damage in an animal model of chronic polyarthritis. Our work paves the way for future studies of inflammatory processes involved in TMJ alterations and the development of innovative therapies for this painful condition in humans, for which the therapeutic options are still very limited.

## Materials and methods

### Mice

K/BxN mice generated as previously described by Kouskoff et al.^10^ spontaneously developed arthritis. KRN transgenic mice were crossed with non-obese diabetic mice (NOD). The resulting K/BxN mice exhibited significant reproducible joint inflammation after 30 days. Control (*n* = 23) and K/BxN (*n* = 67) mice aged 6–8 months were euthanized by intraperitoneal injection of pentobarbital (0.05 mL·g^−1^, Dolethal, Centravet Nancy, France) before decapitation and dissection of the heads and knees for histology, immunofluorescence, micro-computed tomography (μCT), and synoviocyte cultures. Micro-magnetic resonance imaging (μMRI) was performed on euthanized animals. Supplementary Table 2 summarizes the number of mice used for each experiment.

### Ethics statement

Animal experiments were authorized by the “Ministère de l’Enseignement Supérieur et de la Recherche” under agreement number APAFIS #16565-2018083014246137. The local Ethics Committee “Comité Régional d’Ethique en Matière d’Expérimentation Animale de Strasbourg (CREMEAS)” specifically approved this study.

### Histology

The heads and knees were placed in 4% paraformaldehyde (PFA) in PBS and demineralized with Decalcifier II (Leica Microsystems, Nanterre, France) at 37 °C for 3 h under agitation. The heads were cut longitudinally to separate the right and left TMJ before dissection. Coronal and sagittal paraffin sections, 10-μm thick, were stained with alcian blue/fast red and safranin O/fast green. Synoviocytes at passage 3 were fixed with PFA 4% for 10 min at 4 °C and stained with hematoxylin–eosin.

### Immunofluorescence

TMJs were embedded in Tissue Tek® and frozen. Cryostat sections (10 μm) were fixed with 4% PFA for 10 min at 4 °C, saturated with 0.1% Triton X-100 and 1% BSA for 1 h, and then rinsed three times with PBS. The sections were incubated overnight at 4 °C with anti-IL-1β, biotinylated anti-IL-6, anti-osteopontin, anti-bone sialoprotein II (BSPII), anti-Runt-related transcription factor 2 (RUNX2), and anti-CD31 antibodies (See Supplementary Table 3). For anti-Col-I and anti-Col-II antibodies, the sections were fixed with acetone at −20 °C for 10 min, and for the anti-aggrecan antibody, the sections were fixed with methanol at −20 °C for 10 min before saturation with BSA 1% and FBS 10% in PBS for 1 h at room temperature. After three washes with PBS, the sections were incubated for 1 h with secondary antibody (see Supplementary Table 3) (Thermo Fisher Scientific, Illkirch, France) and for 5 min with 200 nmol·L^−1^ 4′,6-diamidino-2-phenylindole (DAPI; Euromedex, Souffelweyersheim, France). The samples were observed under an epifluorescence microscope (Leica DM4000B).

Synoviocytes at passage 3 were fixed with PFA 4% for 10 min at 4 °C, saturated with 0.1% Triton X-100 and 1% BSA for 1 h, and then rinsed three times with PBS. The cells were incubated overnight at 4 °C with an anti-fibronectin, anti-vimentin, anti-CD248 (TEM1), or anti-CD90 antibody (see Supplementary Table 3). After three washes with PBS, some cells were incubated for 30 min with Alexa Fluor 546 phalloidin or with secondary antibodies (Molecular Probes, Thermo Fisher Scientific, Illkirch, France).

### μCT

Half of the heads were fixed with 4% PFA and then immobilized in agarose in a Falcon tube. Tomography experiments were carried out by using the μCT X-ray system nanotom® m (GE Sensing & Inspection Technologies GmbH, Wunstorf, Germany) equipped with a 180 kV–15 W high-power nanofocus tube with a tungsten transmission target. X-ray μCT was performed with an isotropic pixel size of 8 μm and a field of view of approximately 24.6 mm × 19.2 mm.^2^ For each measurement, the sample was irradiated by X-rays with an acceleration voltage of 70 kV and a beam current of 260 mA. At each rotation angle position, four images with an exposure time of 0.5 s were acquired and averaged to a projection. A total of 1 600 projections over 360° resulted in a total scan duration of approximately 67 min.

Data acquisition and reconstruction were performed with Phoenix Datos x 2.0 software (Phoenix X-ray, GE Sensing & Inspection Technologies GmbH, Wunstorf, Germany). After a first step of segmentation, 3D isosurface reconstructions of TMJs were performed to visualize bone defects on both the temporal and mandibular condyles (Microview, Parallax Innovations Inc., Ilderton, Canada).

### Micro-magnetic resonance imaging (μMRI)

Just after lethal injection of pentobarbital, the mouth was kept in a closed position, and mice were placed in an animal cradle. To standardize the position of the condyle, all acquisitions were made with the mouth closed. The MRI exams were performed on a 7 T MRI Biospec 70/30 USR system (Bruker Biospin, Ettlingen, Germany). Transmission was achieved with a quadrature volume resonator (inner diameter of 86 mm), and a 10-mm surface coil placed on the side of the head between the eye and the external auditory meatus closer to the joint was used for signal reception (Bruker BioSpin, Ettlingen, Germany). MRI experiments were analyzed with ParaVision 6.0.1 software. A T2-weighted anatomical dataset was acquired by using a 3D-RARE sequence with a voxel size of 67 μm × 67 μm × 67 µm. The remaining parameters were as follows: matrix = 150 × 150 × 150, TE = 33 ms, TR = 2 s, N avg = 1, RARE-Factor = 14, and acquisition time = 53 min.

### Image processing and analysis

The signal bias of T2-weighted images (T2WIs) induced by the surface coil was corrected with N4 bias correction (Advanced Normalization Tools, ANTs). A portion of the bone was segmented and then used to extract a matrix of rigid registration by using FLIRT (FMRIB Software Library, Oxford, UK). This matrix was applied to the T2WIs with bias correction. An automatic segmentation was applied to discriminate bone and liquid by using FAST (FMRIB Software Library, Oxford, UK). The volume estimation and 3D reconstructions were obtained with 3DSlicer (www.slicer.org).

### FLS isolation and culture

FLSs were prepared from TMJs harvested from control and K/BxN mice as previously described.^36^ Condyles were incubated in 6 mL of culture medium containing 1 mg·mL^−1^ collagenase Type I from *Clostridium histolyticum* (Sigma-Aldrich, France) for 3 h at 37 °C. After centrifugation, the pellet was cultured at 37 °C in 5% CO_2_ in FLS medium (RPMI 1640 Gluta-MAX/Medium 199 (40% each, v/v) (Gibco, Thermo Fisher Scientific, France)) containing 250 ng·mL^−1^ amphotericin B (Fungizone, Gibco, Thermo Fisher Scientific, France), 50 U·mL^−1^ penicillin/streptomycin, and 20% FBS (Dutscher, France). The culture medium was changed twice a week, and the cells were subcultured at 80–90% confluence in FLS medium containing 10% FBS prior to characterization at passage 3. The cells were cultured in a 24-well plate (1 × 10^5^ cells per well). After being allowed to adhere overnight, the cells in half of the wells were treated with 1 μg·mL^−1^ LPS (*Escherichia coli*, strain 0128: B12, Sigma-Aldrich, France) for 24 h.

### IL-6 ELISA

IL-6 levels in the sera and supernatants from cultured FLSs were measured by using a commercially available sandwich ELISA by following the manufacturer’s instructions (R&D Systems, DY406). The data are expressed as pg·mL^−1^. All measurements were made in duplicate.

### RNA extraction, reverse transcription, and real-time PCR

Total RNA was extracted from the samples by using Trizol reagent according to the manufacturer’s protocol (Thermo Fisher Scientific, France). Pretreatment with DNAse I (0.1 mg·mL^−1^) was performed to digest the genomic DNA (Thermo Fisher Scientific, France). The total RNA concentration was quantified by using a Nanodrop 1000 (Thermo Fisher Scientific, France). Reverse transcription was performed by using iScript Reverse Transcription Supermix (Bio-Rad Laboratories, France) according to the manufacturer’s instructions.

mRNA expression of *Il-6*, matrix metalloproteinases *Mmp1* (interstitial collagenase), *8* (neutrophil interstitial collagenase), *9* (gelatinase-B), and *13* (interstitial collagenase-3) and *metallopeptidase inhibitor 1* (*Timp1*) was assessed by using iTAq Universal SYBR Green Supermix (Bio-Rad Laboratories, France). *Gapdh* was used as an endogenous RNA control (housekeeping gene) for all samples. Three independent experiments were analyzed in triplicate. The primer sequences used were as follows: *Il-6*, forward 5′ATGAACAACGATGATGCACTTG3′ and reverse 5′TATCCAGTTTGGTAGCATCCAT3′; *Mmp1*, forward 5′TGCCTAGCCTTCCTTTGCTGTT3′ and reverse 5′CCAGGTATTTCCAGACTGTCTCCA3′; *Mmp8*, forward 5′CCGGAATTGATTGCTTGGTA3′ and reverse 5′CGCCTGAAGACACTTCCATT3′; *Mmp9*, forward 5′CTGTCGGCTGTGGTTCAGT3′ and reverse 5′AGACGACATAGACGGCATCC3′; *Mmp13*, forward 5′TGATGAAACCTGGACAAGCA3′ and reverse 5′TAGATGGGAAACATCAGGGC3′; *Timp1*, forward 5′CGCCTAAGGAACGGAAATTTGCAC3′ and reverse 5′CACAGCCAGCACTATAGGTCTTTG3′; *Gapdh*, forward 5′TGCTGATGCCCCCATGTTCGT3′ and reverse 5′CCAAAGTTGTCATGGATGACC3′

The expression level was calculated after normalization to the expression of the housekeeping gene.

### Statistical analysis

Statistical analyses were performed by using GraphPad Prism 5.0 for Windows (GraphPad Software Inc.). Data distribution was checked, and statistical significance was evaluated by Mann–Whitney U test. All data are expressed as the mean ± SEM. *P* **<** 0.05 was considered statistically significant.

## Supplementary information

Characterization of the K/BxN mice used in this study.

Histology of the temporomandibular joint (TMJ) of a 8-month-old control mouse and of eleven 8-month-old K/BxN mice after alcian blue staining.

Micro-computed tomography (μCT) sections and 3D reconstructions of the condyles of one 8-month-old control mice (a) and five 8-month-old K/BxN mice TMJ (b-f)

Magnetic resonance imaging (MRI) and 3D reconstructions of the TMJ of 3 control mice (a) and of 7 K/BxN mice (b).

Comparative analysis of TMJ damages in various polyarthritis mouse models.

Number of mice used for each experiment.

Primary and secondary antibodies used for indirect immunofluorescence.
