# Supplementary material for: Temporomandibular joint damage in K/BxN arthritic mice
Source: Int J Oral Sci. 2020 Feb 6;12:5. doi: 10.1038/s41368-019-0072-z (PMC7002582; doi:10.1038/s41368-019-0072-z)
Supplement: Supplementary file 1 — Characterization of the K/BxN mice used in this study. [file 41368_2019_72_MOESM1_ESM.docx]

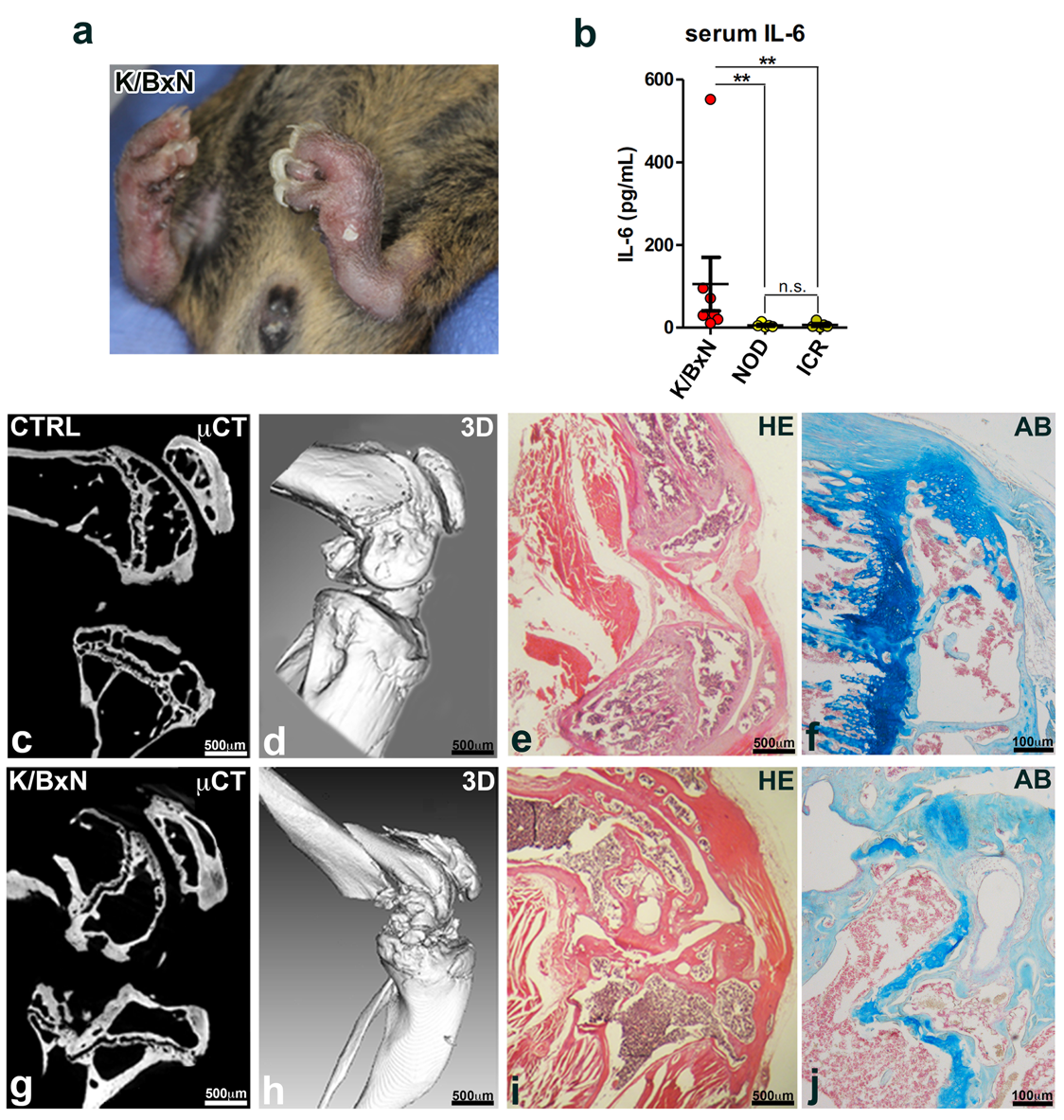


**Supplementary Figure 1.** Characterization of the K/BxN mice used in this study. **(a)** Hind limbs of a 8 month-old K/BxN mouse. **(b)** Quantification by Elisa of IL-6 (pg/mL) in the serum of K/BxN mice (n=7) and of NOD (n=5) and ICR (n=5) control mice. **(c, d, g, h)** Micro-computed tomography sections and 3D reconstruction of a control (c, d) and a K/BxN (g, h) mouse knee. **(e, i)** Hematoxylin-eosin staining and (**f, j)** alcian blue staining of knee sections of a control (e, f) and a 6 month-old K/BxN (i, j) mouse. Values are presented as means ± SD; **P < 0.001. n.s. not significant.
