# Supplementary material for: Temporomandibular joint damage in K/BxN arthritic mice
Source: Int J Oral Sci. 2020 Feb 6;12:5. doi: 10.1038/s41368-019-0072-z (PMC7002582; doi:10.1038/s41368-019-0072-z)
Supplement: Supplementary file 2 — Histology of the temporomandibular joint (TMJ) of a 8-month-old control mouse and of eleven 8-month-old K/BxN mice after alcian blue staining. [file 41368_2019_72_MOESM2_ESM.docx]

**
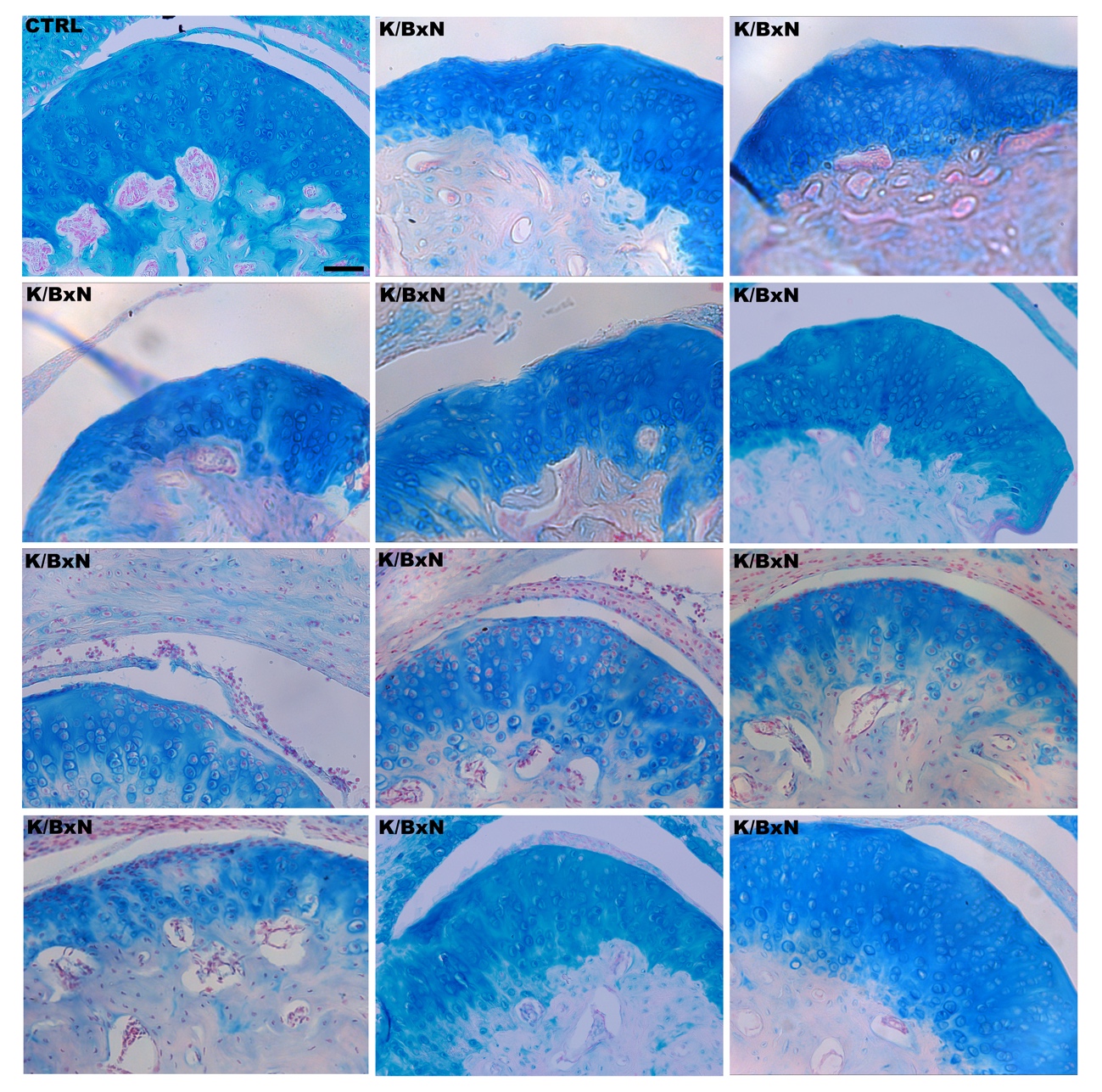
**

**Supplementary Figure 2.** Histology of the temporomandibular joint (TMJ) of a 8 month-old control mouse and of eleven 8 month-old K/BxN mice after alcian blue staining. Histological sections show erosion and a different thickness depending on the mouse. Bar = 50 μm.
