# Supplementary material for: Temporomandibular joint damage in K/BxN arthritic mice
Source: Int J Oral Sci. 2020 Feb 6;12:5. doi: 10.1038/s41368-019-0072-z (PMC7002582; doi:10.1038/s41368-019-0072-z)
Supplement: Supplementary file 3 — Micro-computed tomography (μCT) sections and 3D reconstructions of the condyles of one 8-month-old control mice (a) and five 8-month-old K/BxN mice TMJ (b-f) [file 41368_2019_72_MOESM3_ESM.docx]

_
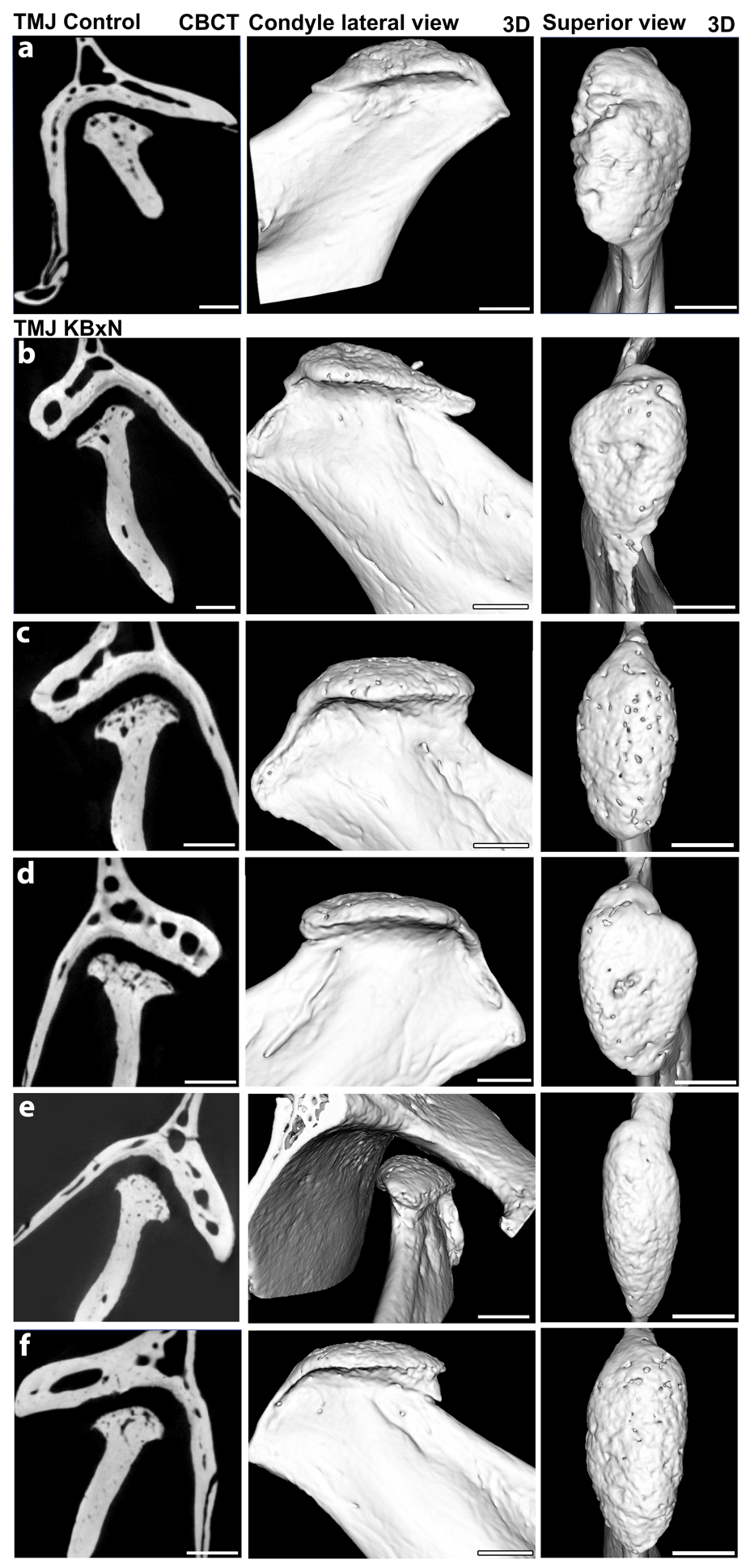
_

**Supplementary Figure 3.** Micro-computed tomography (μCT) sections and 3D reconstructions of the condyles of one 8 month-old control mice (a) and five 8 month-old K/BxN mice TMJ (b-f) to illustrate different bone abnormalities (erosion) that were detected in the TMJ of K/BxN mice. Bars = 0.5mm.
