# Supplementary material for: Temporomandibular joint damage in K/BxN arthritic mice
Source: Int J Oral Sci. 2020 Feb 6;12:5. doi: 10.1038/s41368-019-0072-z (PMC7002582; doi:10.1038/s41368-019-0072-z)
Supplement: Supplementary file 4 — Magnetic resonance imaging (MRI) and 3D reconstructions of the TMJ of 3 control mice (a) and of 7 K/BxN mice (b). [file 41368_2019_72_MOESM4_ESM.docx]

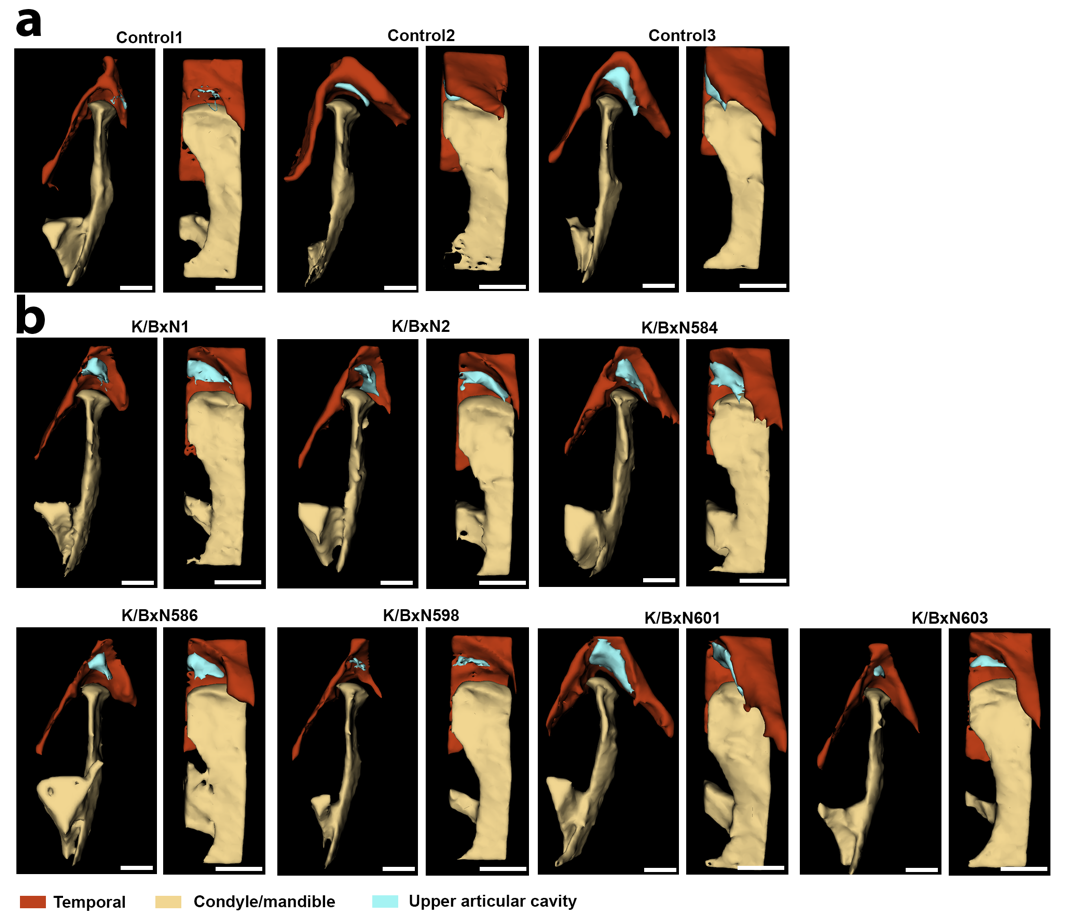


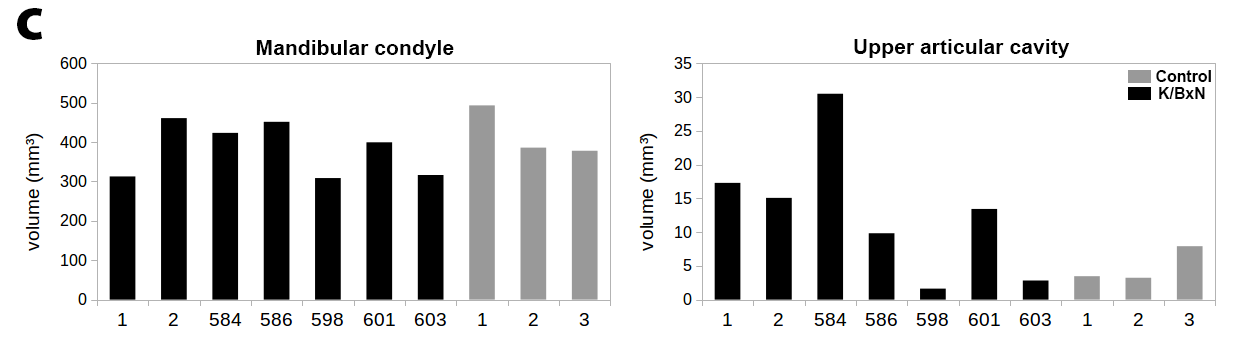


**Supplementary Figure 4.** Magnetic resonance imaging (MRI) and 3D reconstructions of the TMJ of 3 control mice (a) and of 7 K/BxN mice (b). We observed heterogeneous volumes of the upper articular cavity in K/BxN mice, whereas this measure was homogeneous in controls. (c) Histograms showing the volume measurement of the upper articular cavity and of the mandibular condyles of 7 K/BxN mice and of 3 control mice. Bars = 2 mm.
