# Supplementary material for: Temporomandibular joint damage in K/BxN arthritic mice
Source: Int J Oral Sci. 2020 Feb 6;12:5. doi: 10.1038/s41368-019-0072-z (PMC7002582; doi:10.1038/s41368-019-0072-z)
Supplement: Supplementary file 5 — Comparative analysis of TMJ damages in various polyarthritis mouse models. [file 41368_2019_72_MOESM5_ESM.docx]

| **Techniques used** | **K/BxN (present study)** | **Serum induced arthritis**  (Safi et al., 2018) | **TNF-α transgenic mice**  (Dobsak et al., 2017) |
| --- | --- | --- | --- |
| **Histology** | Discontinuity in the fibrous layer and reduced cartilage thickness (67%) | No evidence of inflammatory processes | Cartilage not visible, fibrous tissue invading the joint space (100%) |
| **Micro-CT** | Bone abnormalities like erosion (66%) | No surface erosion observed | Surface erosion, bone volume/tissue volume reduced (100%) |
| **Immunofluorescence** (ColII, ColI, Aggrecan, OPN, CD 31, BSPII, Runx2) | Col-I undetectable in the fibrous layer (70%) | No difference with control for  IL-17, OPG, VEGF | Not tested |
| **Micro MRI** | Increased TMJ articular volume (57%) | Not tested | Not tested |
| **FLS culture** | Aggressive (pro-inflammatory) phenotype (100 %) | Not tested | Not tested |
| **Hind limbs** | Distortions (100%) | Reduced locomotor activity (78%) | Not tested |

**Supplementary Table 1. Comparative analysis of TMJ damages in various polyarthritis mouse models.** BSP : bone sialoprotein, Col : collagen, IL : interleukin, OPG : osteoprotegerin, OPN : osteopontin, VEGF : vascular endothelial growth factor.
