# Supplementary material for: Temporomandibular joint damage in K/BxN arthritic mice
Source: Int J Oral Sci. 2020 Feb 6;12:5. doi: 10.1038/s41368-019-0072-z (PMC7002582; doi:10.1038/s41368-019-0072-z)
Supplement: Supplementary file 6 — Number of mice used for each experiment. [file 41368_2019_72_MOESM6_ESM.docx]

|  | Histology | Micro-CT | Immunofluorescence | RMI | FLS culture |
| --- | --- | --- | --- | --- | --- |
| Control | 5 | 5 | 5 | 3 | 5 |
| K/BxN | 25 | 10 | 15 | 7 | 10 |

**Supplementary Table 2.** Number of mice used for each experiment.
