# Supplementary material for: Temporomandibular joint damage in K/BxN arthritic mice
Source: Int J Oral Sci. 2020 Feb 6;12:5. doi: 10.1038/s41368-019-0072-z (PMC7002582; doi:10.1038/s41368-019-0072-z)
Supplement: Supplementary file 7 — Primary and secondary antibodies used for indirect immunofluorescence. [file 41368_2019_72_MOESM7_ESM.docx]

| **Primary antibodies** | **Species origin** | **Manufacturer** | **Dilution** | **Secondary antibodies** | **Dilution** |
| --- | --- | --- | --- | --- | --- |
| Anti-IL-1β | Rabbit polyclonal | Santa Cruz  Biotechnologies | 1/200 | donkey anti-rabbit Alexa Fluor 594 | 1/500 |
| Biotin anti-IL-6 | Rat  monoclonal | Ozyme | 1/100 | Cy3 streptavidin conjugate | 1/50 |
| Anti-fibronectin | Mouse monoclonal | BD Biosciences | 1/100 | donkey anti-mouse Alexa Fluor 488 | 1/200 |
| Anti-TEM1 | Mouse  monoclonal | Santa Cruz  Biotechnologies | 1/50 | donkey anti-mouse Alexa Fluor 488 | 1/200 |
| Anti-CD90 | Rat  monoclonal | Ozyme | 1/100 | donkey anti-rat Alexa Fluor 594 | 1/500 |
| Anti-vimentin | Rabbit polyclonal | Abcam | 1/200 | donkey anti-rabbit Alexa Fluor 594 | 1/500 |
| Anti-Col I | Rabbit  polyclonal | Sigma | 1/75 | donkey anti-rabbit Alexa Fluor 488 | 1/200 |
| Anti-Col II | Rabbit  polyclonal | Sigma | 1/75 | donkey anti-rabbit Alexa Fluor 488 | 1/200 |
| Anti-osteopontin | Rabbit  polyclonal | Abcam | 1/200 | donkey anti-rabbit Alexa Fluor 488 | 1/200 |
| Anti -RUNX2 | Rabbit  polyclonal | Sigma | 1/100 | donkey anti-rabbit Alexa Fluor 488 | 1/200 |
| Anti-aggrecan | Rabbit  polyclonal | Santa Cruz  Biotechnologies | 1/100 | donkey anti-rabbit Alexa Fluor 488 | 1/200 |
| Anti-CD31 | Rat  monoclonal | BD Pharmingen | 1/100 | donkey anti-rat Alexa Fluor 594 | 1/500 |
| Anti-BSPII | Rabbit  polyclonal | Abcam | 1/200 | donkey anti-rabbit Alexa Fluor 488 | 1/200 |

**Supplementary Table 3.** Primary and secondary antibodies used for indirect immunofluorescence.
